# My Turning Point Tells the Story: A Longitudinal Examination of Greater Episodic Detail and Youth Depressive Symptoms

**DOI:** 10.1007/s10802-023-01096-3

**Published:** 2023-07-28

**Authors:** Laurel Keats, Paul Jose, Karen Salmon

**Affiliations:** School of Psychology, Victoria University of Wellington - Te Herenga Waka, PO Box 600, Wellington, New Zealand

**Keywords:** Depressive symptoms, Adolescence, Autobiographical memory, Episodic detail, Turning point, Self-focus

## Abstract

Although research findings show that the personal memories of people who are depressed are characterized by sparse episodic detail, under some circumstances, the opposite pattern emerges. Specifically, a recent study (Salmon et al., 2021) has shown that for community youth, *greater* episodic detail in a highly self-relevant narrative (a life turning point) predicted increased depressive symptoms concurrently and one year later. In a new longitudinal study of young people (*N* = 320 at Time 1, *M* = 16.9 years; 81% female) followed up over six months, we aimed to replicate and extend this finding. In Study A, we compared the turning point with a narrative about a conflict event, to establish whether the detail in a turning point memory uniquely predicted depressive symptoms. Supporting the first hypothesis, at both time-points, greater episodic detail was concurrently positively associated with depressive symptoms for turning point narratives only. Contrary to our second hypothesis, greater detail did not predict increased depressive symptoms longitudinally. The reverse pattern was significant, however, in that greater initial depressive symptoms predicted greater detail uniquely in the turning point narrative six months later. In Study B, we determined that the concurrent association between episodic detail and depressive symptoms in turning points (but not conflict events) was exacerbated by linguistic markers of self-focus (greater I-talk and lower distancing language). These findings suggest that greater detail in a turning point narrative may uniquely signify risk of psychological distress when youth narrate the experience with heightened self-focus.

Depression typically has its onset during adolescence (Fergusson & Horwood, 2001; Kessler et al., 2007). Even low levels of depressive symptoms during this time can pose a serious risk for future psychological difficulties, and early detection and intervention is key (Balázs et al., 2013; Lee et al., 2019). Adolescence is a critical period of self-concept development (Harter & William, 2016) which may confer risk for the onset of psychological distress (Rapee et al., 2019). Autobiographical memories are intimately connected to self-concept and identity (Conway, 2005; McAdams, 2001) and to psychological wellbeing (Waters, 2014). Autobiographical memory difficulties may therefore provide an early warning sign or even a critical mechanism for developing depression (Dalgleish & Werner-Seidler, 2014).

Research has typically found that people who are depressed, relative to non-depressed people, have reduced ability to recall specific past events (see Hallford et al., 2021 for a meta-analysis) and their personal memories are sparse and impoverished in *episodic detail* (e.g., actions, thoughts, feelings and event-specific information) that locates the experience in time and place (Biedermann et al., 2017; Söderlund et al., 2014). Thus, for both adults and adolescents, memory interventions for depression have emerged that seek to enhance recall of specific and detailed memories (Barry et al., 2021; Hallford et al., 2020). Yet recent findings with community (non clinical) adolescents (Salmon et al., 2021) whose depressive symptoms were beginning to escalate, showed the opposite pattern: *more*—rather than less—episodic detail in a highly self-relevant life *turning point* narrative (an episode that changed who they are and their life; see Grysman & Hudson, 2011; McAdams et al., 2001) concurrently and longitudinally predicted greater depressive symptoms. In contrast, *non-episodic detail* (general information external to the main event) did not.

Considering the intimate connection between autobiographical memories and the self (Conway, 2005; Wilson & Ross, 2003), one possible explanation for the relationship between greater episodic detail and depressive symptoms is excessive self-focus (Kyung et al., 2016; Salmon et al., 2021; Wang et al., 2018), which is perhaps heightened by the high self-relevance of the turning point (Grysman & Hudson, 2011). The attentional bias of *self-focus*—heightened awareness of internal self-referential information – has been robustly implicated in depression (see Mor & Winquest, 2002 for a meta-analysis). Moreover, convergent research suggests that episodic detail may reflect a focus on the self (Wang, 2001, 2013), or an ‘own eyes’ first-person perspective (Akhtar et al., 2017). Theoretically reliving past events in detail is linked to a sense of self as continuous through time (*autonoetic consciousness*; Wheeler et al., 1997) and increased first-person perspective (Zaman & Russell, 2022). Whereas, shifting perspective away from the self decreases episodic detail (King et al., 2022). Additionally, when self-focus is induced, people, particularly when they are depressed, recount the past in greater detail and report increased negative affect (Kross et al., 2012). ‘Stepping back’ and recalling an event with less detail (taking a distant perspective), on the other hand, reduces negative affect (Boucher & Scoboria, 2020; Orvell et al., 2021) and may buffer against escalating depressive symptoms (Kross & Ayduk, 2009; Orvell et al., 2022a).

Therefore, the overarching aim of the two studies reported here (Studies A and B) was to test the role of self-focus on episodic detail and depressive symptoms by adopting two different analytic approaches with the same dataset. In Study A, in a sample of young people (*M* = 16.9 years), followed up over six months, we examine whether, in line with our previous study (Salmon et al., 2021), greater episodic detail (but not non-episodic detail) in a narrative of a life turning point predicted depressive symptoms, both concurrently and longitudinally. Further, we extend previous research to compare the pattern of results for the turning point memory narrative with those of another salient but less self-relevant memory narrative, namely a conflict event, in which young people were not asked to draw an implication for the self. Thus, Study A was designed to test the hypothesis that greater episodic detail in a turning point memory narrative (prompting greater self-focus) would uniquely predict youth depressive symptoms. Study B was designed to extend findings of Study A, by adopting the method of linguistic analyses to assess differences between the two narrative types (turning point and conflict event) and test whether greater detail uniquely in the turning point memory was exacerbated by indices of self-focused attention (greater I-talk and lower distancing). As covariates, we included age, gender and verbal fluency (as a measure of language and executive functioning; Henry et al., 2015; Whiteside et al., 2016), given previous findings demonstrating that these variables may be related to both autobiographical memory and depressive symptoms (Grysman & Hudson, 2013; Salmon et al., 2021).

## Study A: Does Greater Episodic Detail Predict Depressive Symptoms Uniquely in Turning Points?

From a cognitive theoretical perspective (Tulving, 1972), autobiographical memories are comprised of both *episodic* (event details that report the when, who, what and where) and *semantic* information (general facts about the world and the self). A large body of research shows that individuals experiencing or recovering from depression show difficulties in specific memory retrieval (Hallford et al., 2021; Williams et al., 2007). Thus, recently the amount of episodic and non-episodic content in personal memories (Addis et al., 2008; Levine et al., 2002; Miloyan et al., 2019 for a review) has been studied in relation to a range of psychological difficulties. Yet, in regard to depression, findings have been mixed (Kyung et al., 2016; Salmon et al., 2021; Söderlund et al., 2014). This may be in part due to differences in level of depression or memory type. We suggest that one possibility is that when recalling highly self-focused experiences (as in a life turning point narrative), community youth with emerging depressive symptoms may have enhanced, rather than disrupted, access to greater episodic detail.

Turning point memory narratives explicitly prompt for self-reflection and a resolution (how the event changed the person and their life) and are considered uniquely central to the life story (the personalised, internalised and dynamic sense of self) that is drawn from an individual’s most meaningful past experiences (McAdams, 2001). As turning points describe significant personal change, they tend to be, for both adolescents and adults, more complex and insightful than other life narratives and non-life story memories (Grysman & Hudson, 2010). Turning points have also been reported as more self-relevant than memories primed by a questionnaire about the self (Grysman & Hudson, 2011). Not surprisingly, turning point narratives are therefore rated as more central to identity than memories of transitions (e.g., a shift in external circumstances; Enz & Talarico, 2016), and longitudinal research shows that differences in the ways that they are understood and interpreted (adaptive self-reflection) is related to psychological well-being for youth (Tavernier & Willoughby, 2012). Thus, we suggest that episodic detail may uniquely predict depressive symptoms in turning points as they elicit self-focus, whereas another salient life experience (such as a conflict event) is less likely to do so.

## Overview of Study A

In Study A, we tested our proposal that greater episodic detail in a highly self-relevant turning point memory narrative uniquely predicts depressive symptoms. Therefore, we compared results between two event types: a narrative of turning point and its resolution (an implication for the self) and another salient life event, a conflict event, which did not include an explicit instruction to provide implications for the self. Thus, in Study A, we investigated whether episodic detail was positively associated, concurrently and longitudinally, with adolescents’ depressive symptoms uniquely in turning point memory narratives. Based on our previous research (Salmon et al., 2021), we hypothesised that greater episodic detail (but not non-episodic) detail in a turning point would be positively associated with depressive symptoms concurrently (Hypothesis 1); and moreover, predict an increase in depressive symptoms across six months (Hypothesis 2), whereas both the amount of episodic and non-episodic detail in a conflict event memory narrative would not be significantly related to depressive symptoms (Hypothesis 3).

## Method

### Participants

Participants were 320 (259 females, 59 males, one “other”, and one did not include gender) adolescents from nine schools across New Zealand/Aotearoa. The mean age was 16.9 years (*SD* = 0.59), and did not differ by gender. Participants’ self-identified ethnicity was 74.3% NZ European/Pakeha; 5.5% Pasifika; 5.8% Asian; 11.9% Māori; and 2.3% “other”. Most (85%) of participants came from schools within the top half of socio-economic communities, whereas 15% came from schools from the bottom half based on decile ratings (www.parent.education.govt.nz).

At the follow-up data collection, some participants (*N* = 130) were not in attendance at school or opted not to take part in the research. There was no significant difference in depressive symptoms between completers (*M* = 7.75) and non-completers (*M* = 7.66), *t* = -0.18, *p* = 0.86, 95% CI = [-1.01, 0.85]. Young people who left the study were significantly older (*M* = 204.18 months) than those who completed the study (*M* = 202.43 months) *t* = 2.21, *p* = 0.03, 95% CI = [0.19, 3.31] and exhibited lower verbal fluency (*M* = 27.68) compared to completers (*M* = 29.73) *t* = -2.54, *p* = .01, 95% CI = [-3.64, -0.46]. These variables were entered as covariates in all analyses.

### Declarations

At the beginning of the data collection session, written consent was obtained from each participant age 16 years and older. For participants younger than 16 years, information about the study was sent to parents, and if they had provided consent, youth written assent was obtained at the beginning of the session. The young people received a small gift (pizza, chocolate bar) in appreciation of their participation. Ethical approval was granted by the School of Psychology Ethics Committee under delegated authority of the Human Ethics Committee. The authors declare no conflict of interests.

### Measures

In line with Salmon et al. (2021), each participant was given an answer booklet containing a range of measures to be completed in written form. Only measures directly relevant to the hypotheses of the current study are included here.

#### Depressive Symptoms

The 12-item Children’s Depression Inventory 2^nd^ Edition – short form (CDI 2:SR[S]; Kovacs, 1992) measured depressive symptoms. Participants responded on a scale of one to three to describe symptom severity over the past week. The 12 items were summed to produce total scores (maximum = 24). Internal consistency was 0.83 at Time 1, 0.86 at Time 2.

#### Verbal Fluency

The sum scores of letter and category fluency tasks (Cohen & Stanczak, 2000) controlled for the potential influence of ease of language processing (Whiteside et al., 2016) and executive functioning (Henry et al., 2015). Letter fluency involved writing down as many words as possible (within one min) that began with the given letter (S), not including people or places, or the same word but with a different ending. Category fluency similarly involved writing as many words as possible that belonged to the category of animals.

#### Narrative Task

A modified version (following Reese et al., 2017; Salmon et al., 2021) of the guided autobiography memory task (McAdams et al., 2006) was adopted to prompt turning point and conflict event narratives.

Instructions for the turning point: 

*I would like you to think back over your life and identify a past event that has changed your life or the kind of person you are. It could be something from any area of your life – your relationships with other people, your work and school, your outside interests, and so forth. Please identify a particular episode in your life story that you now see as a turning point in what your life is like or what you are like as a person. Please describe what happened, when it happened, who was involved, what you were thinking and feeling, why this experience is significant, and how it changed your life or you as a person.*

Instructions for the conflict event were similar, but did not include a resolution for the self (how it changed you as a person):* “I would like you to think of a past conflict that you experienced with someone. Try to remember a specific experience in which you had a disagreement with someone, such as a time when you might have argued or had a dispute over something. Please describe what happened, when it happened, who was involved, what you were thinking and feeling and why this experience is significant.*

### Procedure

Responses to the answer booklets were obtained at two time points (six months apart), in group sessions overseen by research assistants. Following the methods of Salmon et al. (2021), participants provided written narratives and completed the questionnaires on-site at school in small groups with research assistants present. Measures were presented in fixed order and the questionnaire remained identical at Time 2; participants were therefore free to recall the same or a different event. Due to unforeseen circumstances, the order of narratives was not fully counterbalanced, with the result that most of the participants (73%) completed the conflict event first at both timepoints and the remaining participants completed the turning point first. Therefore, to account for order effects, we included narrative order as a covariate in all analyses (see below). To summarise, however, narrative order was not a significant covariate in any analysis conducted.

Before beginning data collection, the research assistants informed the class about the aim of the study and began by reading the instructions for the memory narrative aloud while the participants read the same instructions on their own answer booklet. Participant were given five minutes to write each narrative, after which they individually and at their own pace completed the broad suite of self-report measures (including the CDI 2:SR[S]; Kovacs, 1992). Next, the research assistant gave instructions for the verbal fluency tasks involving a one-minute letter and category test (Cohen & Stanczak, 2000).

### Scoring

#### Internal and External Detail

All narratives were transcribed verbatim and coded for amount of episodic (*internal*) and non-episodic (*external*) detail (Addis et al., 2008). Firstly, the main event was isolated, and secondly, each clause was scored as internal (the main event details) or external (other details). Internal details of the main event were coded under five sub-categories: *event details* (who, what, and the actions and reactions of the people involved), *place details* (where), *time details* (when), *perceptual details* (such as visual, olfactory, auditory details) and *emotion/thought details* (the thoughts and feelings of the person at the time of the event). External details were coded as five sub-categories: *semantic* (general knowledge or facts), *repetition* (of prior details), *other* (metacognitions and inferences), *external event detail* (secondary episodic details that are not part of the main event), *external generic* (routines or repeated events). For each, the five categories were summed to provide a total score, one for *internal detail* and one for *external detail*. For example, internal detail was coded as: “I argued (event) with mum (event) yesterday (time) at home (place). I felt angry (emotion). External detail was coded as: We argue often (generic) she has a temper (semantic) I remember (other) another fight recently (external event).

Two trained raters independently coded the narratives. The primary rater coded 100% of the narratives and the secondary rater independently coded a random selection of narratives (25%). ICC reliability using Consistency Single Measures for turning point narratives was 0.77 for total internal and 0.82 for total external detail at Time 1 and 0.82 for total internal detail and 0.86 for total external at Time 2. For conflict event narratives, ICC reliability was 0.92 for total internal detail and 0.83 for total external detail at Time 1 and 0.93 for total internal detail and 0.82 for total external detail at Time 2. The primary rater’s coding was used for all analyses.

## Results

### Preliminary Analyses

The dataset (Time 1 and Time 2 combined) was analysed for patterns of missingness using Little’s MCAR test (Little & Rubin, 2002); results indicated that data were missing in a random fashion, χ2(6873, *N* = 320) = 6861.65, *p* = 0.536. Overall missingness was 21.3% so estimation maximization (EM) was performed to increase statistical power and to minimise bias (Graham, 2009). In SPSS, the missing values were imputed for the scale variables (total internal and external detail, depressive symptoms, and verbal fluency) using EM with 50 iterations.

We are confident that this procedure is consistent with best practices in treating missing values and, importantly, that the amount of data imputed overall falls within recommended guidelines. Research shows that list-wise deletion, i.e., removing participants who miss a timepoint of measurement, introduces bias and reduces generalizability (Jelicic et al., 2010). We employed EM imputation, an iterative algorithm to derive maximum likelihood (ML) estimates, that has been shown to reliably converge in under 50 iterations (Yuan et al., 2012). The ML-based approach is highly recommended for missing data (Schafer & Graham, 2002). Furthermore, EM is recognized as a valid method for this type of imputation (Molenberghs & Verbeke, 2005) and produces reliable estimates at large percentages of missingness (i.e., up to 50%) when data are missing in a random way (Dong & Peng, 2013; Scheffer, 2002). As is typical, we have ensured that the imputation included all available participant data for the measures being studied as well as auxiliary variables at both timepoints (chosen on the basis that they share variance with the imputed variables) to enable more accurate estimation of the missing values (Baraldi & Enders, 2010).

### Descriptive Statistics

The means and standard deviations for each timepoint are presented in Table 1. As expected for a community sample, the mean depressive symptoms scores were relatively low overall (CDI-2:SR[S] scores ranging between zero and 23). Further, and unexpectedly, there was a significant *decrease* in mean depressive symptoms from Time 1 to Time 2 *t*(316) = 4.27, *p* < 0.001. The total amount of internal detail (summed across subcategories) reported in the narratives was also significantly lower at Time 2 than at Time 1 for both the turning point *t*(312) = 5.41, *p* < 0.001, and the conflict event* t*(315) = 3.82, *p* < 0.001, whereas there was no significant difference in external detail. Total internal detail was significantly higher in conflict events compared to turning points at both Time 1 *t*(312) = 11.50, *p* < 0.001, and Time 2 *t*(314) = 15.63, *p* < 0.001. Moreover, total external detail was significantly higher for turning points than conflict events at Time 1 *t*(315) = 11.12, *p* < 0.001, and at Time 2 *t*(315) = 15.40, *p* < 0.001.

**Table 1:** Descriptive Statistics at Time 1 and Time 2

	Mean T1	SD T1	Mean T2	SD T2
Age	16.93	7.10	17.25	7.02
Verbal Fluency	28.90	7.16	30.42	6.15
Depressive symptoms	7.71	4.14	7.13	3.81
Turning Point				
Total Internal Detail	10.55	6.96	8.30	5.42
Total External Detail	9.62	6.05	9.23	4.40
Conflict Event				
Total Internal Detail	17.84	10.06	15.59	7.35
Total External Detail	5.49	4.70	5.26	3.33

Verbal fluency is letter and category fluency, summed (Cohen & Stanczak, 2000). Depressive symptoms were assessed with the Children’s Depression Inventory 2^nd^ Edition – short form (CDI 2:SR[S]; Kovacs, 1992). Total internal detail and total external detail are summed across categories (Addis et al., 2008).

### Correlations Amongst Key Variables

Pearson’s correlations within and across time points are reported for both turning points and conflict events (Table 2). For turning point narratives, Time 1 depressive symptoms were significantly positively correlated with total internal detail at both Time 1 and Time 2, and Time 2 depressive symptoms were significantly positively correlated with total internal detail at Time 2 only. In contrast, for conflict events, within and across timepoints, there were no significant correlations identified between total internal detail and depressive symptoms. There were significant intercorrelations amongst the covariates (age, gender, and verbal fluency).

**Table 2:** Bivariate Correlations Within and Across Time 1 and Time 2

Variable	2	3	4	5	6	7	8	9	10	11	12	13	14
1 Age	-0.03	-0.04	-0.15**	0.03	-0.01	-0.01	-0.04	-0.02	-0.10	-0.13*	-0.04	0.02	-0.02
2 Gender (Female)	-	0.21**	0.16**	0.09	0.27**	0.28**	0.21**	0.26**	0.13*	0.04	0.28**	0.26**	0.21**
3 T1 Verbal fluency		-	-0.15**	0.20**	0.12*	0.23**	0.18**	0.68**	-0.07	-0.06	0.16**	0.17**	0.17**
4 T1 Depression			-	0.12*	0.01	0.02	0.07	-0.06	0.81**	0.25**	-0.01	-0.01	0.06
5 T1 TP Internal				-	-0.05	0.15**	0.14*	0.05	0.11	0.29**	0.10	0.13*	0.14*
6 T1 TP External					-	0.25**	0.26**	0.20**	0.05	0.13*	0.52**	0.24**	0.17**
7 T1 Conflict Internal						-	-0.03	0.25**	0.07	0.08	0.36**	0.34**	0.23**
8 T1 Conflict External							-	0.23**	0.13*	0.08	0.23**	-0.02	0.23**
9 T2 Verbal Fluency								-	-0.05	-0.05	0.25**	0.16**	0.13*
10 T2 Depression									-	0.17**	0.05	0.06	0.06
11 T2 TP Internal										-	-0.09	0.18**	0.05
12 T2 TP External											-	0.25**	0.33**
13 T2 Conflict Internal												-	-21**
14 T2 Conflict External													-

*p < 0.05; **p < 0.01. Gender is coded 0 = male, 1 = female. Verbal fluency is letter and category fluency, summed (Cohen & Stanczak, 2000). Depression was assessed with the Children’s Depression Inventory 2^nd^ Edition – short form (CDI 2:SR[S]; Kovacs, 1992). TP is turning point memory narratives. Total internal detail and total external detail are summed across categories (Addis et al., 2008)

### Data Analytic Strategy for Testing Hypotheses

The hypotheses were tested with multiple hierarchical regressions using SPSS. The covariates of age, gender, verbal fluency and narrative order were entered at Step 1, and then the predictors of total internal detail and total external detail were entered at Step 2 on the dependent variable of depressive symptoms.

### Internal and External Detail: Cross-Sectional Findings

#### Turning Point Narrative

As predicted, at Time 1, total internal detail was a significant positive predictor of depressive symptoms (*β* = 0.14, 95% CI = [0.02, 0.15]), accounting for 2% unique variance over and above the covariates, whereas total external detail was not a significant predictor (*β* = *-*0.01, 95% CI = [-0.08, 0.07]). A parallel hierarchical regression tested the same cross-sectional relationship at Time 2. As predicted, results showed that internal detail was a significant positive predictor of depressive symptoms (*β* = 0.14, 95% CI = [0.02, 0.14]) over and above covariates (again, significantly accounting for 2% unique variance), whereas external detail was not a significant predictor (*β* = 0.05, 95% CI = [-0.06, 0.14]).

#### Conflict Event Narrative

Parallel analyses with the conflict event narrative showed that at both time-points neither total internal detail nor total external detail was a significant concurrent predictor of depressive symptoms: Time 1 total internal detail (*β* = 0.02, 95% CI = [-0.04, 0.06]); Time 1 total external detail (*β* = 0.07, 95% CI = [-0.04, 0.16]; Time 2 total internal detail (*β* = 0.08, 95% CI = [-0.02, 0.11]); Time 2 total external detail (*β* = 0.07, 95% CI = [-0.06, 0.21]). As predicted, the association between episodic detail and depressive symptoms appears to be unique to the turning point narrative.

### Internal and External Detail: Longitudinal Findings

#### Turning Point Narrative

In contrast to our prediction, we found no significant longitudinal association between Time 1 total internal detail (*β* = -0.01, 95% CI = [-0.04, 0.04]) or total external detail (*β* = 0.06, 95% CI = [-0.01, 0.08]) with residualised Time 2 depressive symptoms. Hypothesis 2, therefore, was not supported.

Considering the findings that internal detail was significantly positively associated with depressive symptoms concurrently, and that depressive symptoms decreased across the six-month period (in contrast to our earlier study, Salmon et al., 2021, which found increasing depressive symptoms across one year), we conducted a post-hoc analysis of the longitudinal relationship between depressive symptoms at Time 1 and residualised total internal detail at Time 2 (see Table 3). Results showed that Time 1 depressive symptoms significantly predicted increased total internal detail at Time 2 (*β* = 0.19, 95% CI = [0.10, 0.39]), accounting for 3% unique variance.

**Table 3:** Hierarchical Regression of Time 1 Depressive Symptoms Predicting Time 2 Total Internal Detail for Turning Point Narratives

		*B*	*SE*	*β*	*R*^2^	∆*R*^2^
Step 1	(Constant)	27.15	8.69		0.12**	
	Narrative order	-1.39	0.72	-0.11		
	Gender	0.73	0.80	0.05		
	Age	-0.09	0.04	-0.12*		
	T1 Verbal fluency	-0.09	0.04	-0.12*		
	T1 Internal detail	0.23	0.04	0.29**		
Step 2	(Constant)	20.72	8.77			
	T1 Depressive Symptoms	0.24	0.07	0.19**	0.14	0.03**

*p < 0.05; **p < 0.01. *N* = 310

#### Conflict Event Narrative

Parallel analyses established that neither total internal detail (*β* = 0.07, 95% CI = [-0.002, 0.05]) nor total external detail (*β* = 0.05, 95% CI = [-0.01, 0.10]) in the conflict event narratives significantly predicted depressive symptoms at Time 2. Moreover, greater depressive symptoms at Time 1 did not predict an increase in total internal detail at Time 2 (*β* = *-*0.03, 95% CI = [-0.25, 0.14]).

### Study A Discussion

The overarching aim of Study A was to test the hypothesis that greater episodic (internal) detail would concurrently and longitudinally predict depressive symptoms in the turning point memory narrative only, and not the conflict memory narrative. Our key predictions were partially supported. Consistent with hypothesis one, youth with elevated levels of depressive symptoms concurrently recalled turning points (but not conflict events) with greater episodic detail (but not greater non-episodic detail). Although overall episodic detail explained a relatively modest contribution to unique variance in depressive symptoms (2%), the relationship remained after controlling for important covariates (age, gender, verbal fluency, narrative order and external detail) and replicates previous research (Salmon et al., 2021).

In contrast, the second hypothesis, that episodic detail would predict increased depressive symptoms over time, was not supported. Yet post hoc analyses showed a significant positive association in the opposite temporal direction: youth with greater depressive symptoms at Time 1 recalled turning points (but not conflict events) with increased episodic detail at Time 2. Therefore, the longitudinal association is similarly unique for turning point memories.

Drawing on a raft of research findings suggesting a link between remembering past events in detail and self-focus (Kross et al., 2012; Wang, 2001; Zaman & Russell, 2021), we propose that, for the youth in our study whose depressive symptoms are emerging, their turning point narratives may contain greater episodic detail due to heightened self-focus that has been demonstrated to be robustly associated with negative affect and depression (Mor & Winquest, 2002). In sum, greater episodic detail may be associated with depressive symptoms in turning points (but not in conflict event narratives) due to the increased salience of the self during recall (Grysman & Hudson, 2011) eliciting unhelpful and excessive self-focused attention.

### Study B: Self-Focused Linguistic Markers in Relation to Greater Episodic Detail and Depressive Symptoms

Following our findings from Study A, we undertook a follow-up analysis to systematically test whether excessive self-focus might account for the unique association between episodic detail and depressive symptoms in turning point memory narratives. One valid method of examining self-focused attention utilises the Linguistic Inquiry and Word Count programme (LIWC; Pennebaker et al., 2001). Greater proportion of first-person pronouns (i.e., I, me, my), known as *I-talk*, indicates self-focused perspective (Orvell et al., 2022b) and is consistently linked to depression (Edwards & Holtzman, 2017; Tackman et al., 2019). Along the same lines, but in the opposite direction, research shows that linguistic psychological *distancing* (i.e., greater use of past/future tense and second/third person pronouns) is an indication of *less* self-focused perspective. Reflecting on the past with this self-distancing approach reduces negative affect for youth and adults (Nook et al., 2017, 2020; Orvell et al., 2021) and improves depression trajectories (Cohen et al., 2022; Orvell et al., 2022a). Therefore, the current study investigates both I-talk and distancing as linguistic measures of the extent to which youth may have taken a self-focused perspective in both event types (turning point and conflict memory narratives).

Despite research linking remembering the past in greater episodic detail to self-focus (Akhtar et al., 2017; King et al., 2022), no research to our knowledge has investigated how linguistic markers of self-focus relate to both depressive symptoms and the amount of episodic detail in autobiographical memories. Thus, following the findings from Study A, we preregistered (https://osf.io/96f7q/) an extension of the above findings using the LIWC linguistic analysis programme. First, we proposed to test the hypothesis that linguistic markers of self-focused perspective would be positively associated with both greater episodic detail and depressive symptoms. Second, we posed a research question as to whether, as for Study A, associations might differ by event type (turning point and conflict event). Finally, to test our proposal that heightened self-focus may play a role in greater episodic detail and depressive symptoms, we examined whether the association would be exacerbated by the linguistic indicators of self-focused perspective (higher proportion of I-talk and lower distancing).

## Method

### Linguistic Inquiry and Word Count (LIWC)

Narratives (turning point and conflict event) were analysed individually by the Linguistic Inquiry and Word Count (LIWC-22; Pennebaker et al., 2001) to calculate proportion of *first-person pronouns* (I, me, my, mine). Moreover, an additional measure that was not pre-registered, the composite linguistic *distancing* variable (with lower amounts indicating more focus on the self in the here and now) was included as a second measure of self-focus. The linguistic distancing variable was computed following previous research (Nook et al., 2017, 2020), by averaging z-scores of *articles* (i.e., the, a, an), *large words* (more than seven letters), *first-person pronouns* (reversed), *present focus* (reversed; i.e., present-tense verbs), and *discrepancy words* (reversed; i.e., would, could, should).

## Results

### Preliminary Analyses

Due to some participants not attending school at Time 2, LIWC data were analysed for patterns of missingness using Little’s MCAR test (Little & Rubin, 2002); overall missingness was 28.1% and results did not suggest that data were missing in any non-random way, χ^2^ (1322, *N* = 319) = 1404.52, *p* = 0.06. In SPSS, the missing data for Time 2 linguistic markers were imputed using Estimation maximization (EM) with 50 maximum iterations (including depressive symptoms, age, verbal fluency at both Time 1 and Time 2 as predictors) to increase statistical power and to minimise bias (Graham, 2009). Significant outliers were examined and across the eight variables, and a total of 20 extreme scores (greater than three standard deviations from the mean) were excluded.

### Descriptive Statistics

The means, standard deviations are presented in Table 4. The total proportions of all linguistic features reported in the narratives remained the same from Time 1 to Time 2, except that distancing was significantly lower in the conflict event at Time 2 *t*(289) = 10.45, *p* < 0.001. First-person pronouns were significantly higher in turning points compared to conflict events at Time 1 *t*(291) = 3.76, *p* < 0.001, and Time 2 *t*(315) = 4.74, *p* < 0.001. There was no significant difference in distancing language at Time 1, but at Time 2 greater distancing was observed in the turning point than the conflict event *t*(113) = 15.47, *p* < 0.001.

**Table 4:** Descriptive Statistics at Time 1 and Time 2

	Mean T1	SD T1	Mean T2	SD T2
Turning Point				
I-talk	11.91	3.62	11.63	2.67
Distancing	0.03	1.91	0.09	1.61
Conflict Event				
I-talk	10.87	3.80	10.44	3.23
Distancing	0.02	2.13	-1.52	1.64

*p < 0.05, **p < 0.01. Narratives were scored for I-talk (total proportion of first-person pronouns) and total proportion of Distancing language (Nook et al., 2017, 2020) using Linguistic Inquiry and Word Count programme (LIWC-22; Pennebaker et al., 2001).

**Table 5:** Bivariate correlations within and across Time 1 and Time

Variable	2	3	4	5	6	7	8	9	10	11	12	13	14
1 T1 Depression	0.12*	0.02	0.09	-0.06	0.04	-0.06	0.81**	0.25**	-0.01	0.11	-0.14*	-0.14*	-0.03
2 T1 TP Internal		0.15**	-0.05	0.07	0.01	-0.12	0.11	0.29**	0.13*	-0.05	0.03	0.01	-0.05
3 T1 Conflict Internal			0.00	0.01	-0.03	0.18**	0.07	0.08	0.34**	-0.03	0.06	-0.05	0.02
4 T1 TP I-talk				-0.55**	0.08	-0.04	0.10	0.07	-0.00	0.09	-0.07	-0.02	-0.03
5 T1 TP Distancing					-0.01	0.09	-0.03	-0.04	-0.08	-0.07	0.18**	0.03	0.09
6 T1 Conflict I-talk						-0.58**	0.05	0.01	0.11	0.13*	-0.01	0.08	-0.01
7 T1 Conflict Distancing							-0.05	-0.03	0.02	-0.11	0.09	-0.02	0.05
8 T2 Depression								0.17**	0.06	0.05	-0.11*	-0.14*	-0.01
9 T2 TP Internal									0.18**	-0.01	0.09	-0.05	-0.00
10 T2 Conflict Internal										0.05	-0.05	0.03	0.01
11 T2 TP I-talk											-0.57**	-0.15**	0.10
12 T2 TP Distancing												0.16**	0.29**
13 T2 Conflict I-talk													-0.54**
14 T2 Conflict Distancing													

*p < 0.05, **p < 0.01. Depression was assessed with the Children’s Depression Inventory 2^nd^ Edition – short form (CDI 2:SR[S]; Kovacs, 1992). TP is turning point memory narratives. Total internal detail and total external detail are summed across categories (Addis et al., 2008). I-talk (total proportion of first-person pronouns) and total proportion of Distancing language (Nook et al., 2017, 2020) were scored using Linguistic Inquiry and Word Count programme (LIWC-22; Pennebaker et al., 2001).

### Correlations Amongst Key Variables

Pearson’s correlations were examined between linguistic features (total proportion of I-talk and distancing), internal detail and depressive symptoms within and across each time-point (see Table 5). In contrast to the first hypothesis, results showed that for turning points, the proportion of I-talk was not significantly correlated with total internal detail or depressive symptoms. As expected, however, proportion of distancing in the turning point at Time 2 was negatively correlated (albeit to a small degree) with depressive symptoms at both time-points. Results for the conflict event, in contrast, showed that I-talk at Time 2 was negatively correlated with depressive symptoms at both time-points. In both the turning point and the conflict event there were no significant associations between the linguistic measures of self-focus (I-talk and distancing) and total internal detail. Finally, the measures of self-focus (I-talk and distancing) were negatively correlated at moderate to high levels.

## Turning Point: Does I-talk Exacerbate Internal Detail and Depressive Symptoms

To further investigate the research question we posed, namely that excessive self-focus may play a role in the relationship of episodic detail to depressive symptoms uniquely in turning points, moderation analyses were performed using multiple hierarchical regressions. Covariates (age, gender, verbal fluency and narrative order) were entered at step 1, total internal and external detail at Step 2, the linguistic measure of self-focus (I-talk) at Step 3, and the interaction term at Step 4. Bootstrapping (a nonparametric, resampling approach) was undertaken due to a slight positive skew in the distribution of the interaction terms at both time-points.

**Fig. 1:**
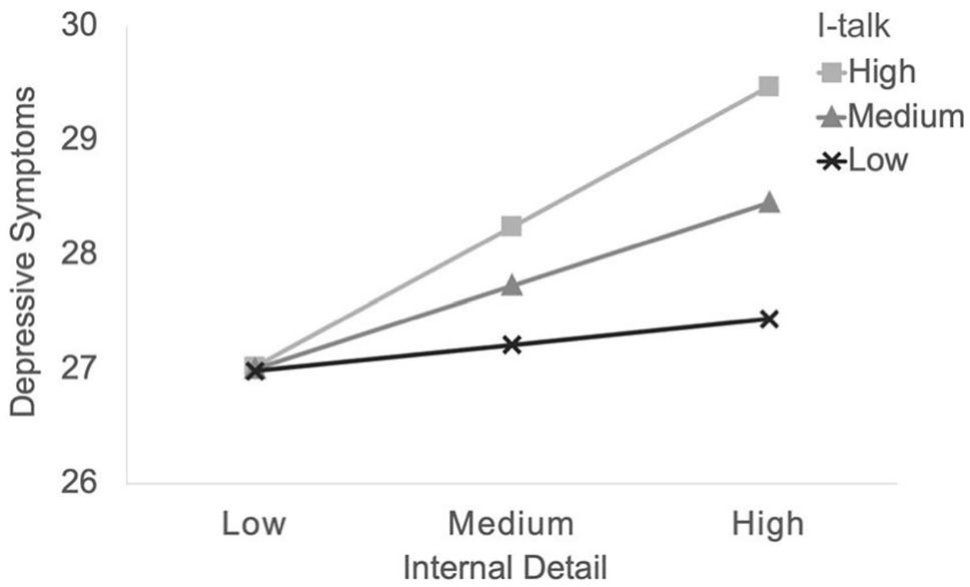
Time 1 Internal detail Moderated by I-talk and Concurrent Depressive Symptoms

Longitudinally the interaction (internal detail X I-talk) was found to not predict depressive symptoms at Time 2 (*β* = 0.07, *p* = 0.60), and the opposite direction (Time 1 depressive symptoms X Time 2 I-talk) predicting greater detail at follow-up did not reach significance (*p* = 0.08). Moreover, concurrently a significant interaction was found between proportion of first-person pronouns (I-talk) and total internal detail predicting depressive symptoms at Time 1 (*B* = 0.02*, SE* = 0.01, *95% CI* = [0.0001, 0.04]),* p* < 0.05). The interaction was graphed, as recommended for interpretation, using the graphing software ModGraph (Jose, 2013) and simple slope statistics were computed for three conditions: low I-talk, slope = 0.03, *t*(296) = 0.59, *p* = 0.56; medium I-talk, slope = 0.11, *t*(296) = 1.94, *p* < 0.05; and high I-talk, slope = 0.18, *t*(296) = 2.44, *p* < 0.05. Figure 1 shows that, I-talk did moderate the relationship between internal detail to depressive symptoms. Results at Time 2 confirmed that high levels of I-talk exacerbate the association: (*B* = 0.04*, SE* = 0.02, *95% CI* = [0.01, 0.08]),* p* < 0.01). As above, slope statistics were computed for the three conditions: low I-talk, slope = -0.02, *t*(306) = -0.26, *p* = 0.80; medium I-talk, slope = 0.10, *t*(306) = 1.74, *p* = 0.08; and high I-talk, slope = 0.21, *t*(306) = 2.83, *p* < 0.01. These slopes show that at both time-points the relationship between total internal detail and depressive symptoms was significant under conditions of high (and medium at Time 1 only) levels of I-talk, but the relationship was attenuated when I-talk was low.

## Turning Point: Does Distancing Buffer Internal Detail and Depressive Symptoms

To further investigate the same question as above, the composite linguistic marker distancing was used to test whether *less* focus on the self in the here and now buffered the maladaptive role of greater internal detail. In the same manner as above, a moderation analysis was performed with distancing at step 3 and the interaction (internal detail X distancing) at Step 4.

**Fig. 2:**
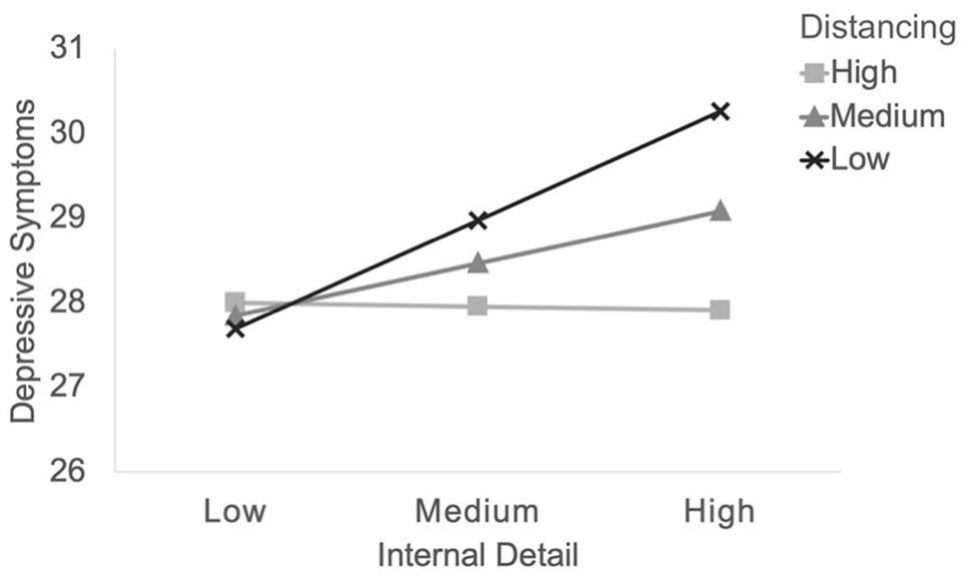
Time 1 Internal Detail Moderated by Distancing and Concurrent Depressive Symptoms

As above, longitudinally the interaction (internal detail X distancing) did not predict depressive symptoms at Time 2 (*β* = 0.02, *p* = 0.77), and the interaction (Time 1 depressive symptoms X Time 2 distancing) predicting greater internal detail at Time 2 did not reach significance (*p* = 0.09). However, concurrently a significant interaction was found between proportion of distancing and total internal detail (*B* = -0.05*, SE* = 0.02, *95% CI* = [-0.09, -0.01]), *p* < 0.01). ModGraph (Jose, 2013) simple slope statistics were computed for three conditions: low distancing, slope = 0.19, *t*(293) = 3.54, *p* < 0.001; medium distancing, slope = 0.09, *t(*293) = 2.85, *p* < 0.01; and high distancing, slope = -0.01, *t*(293) = -0.14, *p* = 0.89. Figure 2 shows that distancing buffered the relationship of internal detail on depressive symptoms.

Results at Time 2 confirmed the same pattern (*B* = -0.07*, SE* = 0.03, *95% CI* = [-0.13, 0.003]). As above, slope statistics were very similar. These slopes show that at both time-points the relationship between total internal detail and depressive symptoms was significant under conditions of low and medium levels of linguistic distancing, whereas a high proportion of distancing provided a buffer.

### Conflict Event: Does Self-Focus Moderate Internal Detail and Depressive Symptoms

In the same manner as above, moderation analyses were performed for the conflict event narratives. No significant concurrent or longitudinal results were found. In sum, self-focus (I-talk or distancing) was not a significant moderator of internal detail and depressive symptoms in conflict event memory narratives.

## Study B Discussion

In Study B, we addressed, first, how linguistic markers of self-focus (higher proportion of I-talk and lower distancing) in the personal memories of young people, in combination with episodic (internal) detail, predicted depressive symptoms across six months. Our hypothesis that heightened self-focused perspective (reflected in I-talk) would be correlated with both greater episodic detail and depressive symptoms was not supported. Yet, results showed that linguistic markers of self-focus (I-talk and distancing) did provide important insights into differences in the ways that turning points and conflict event narratives are recalled. As expected, the turning points yielded overall significantly higher I-talk than the conflict events, supporting the increased salience of the self during recall of the former type. Furthermore, distancing in the turning point was, as expected, negatively correlated with depressive symptoms. Yet, associations between indices of self-focus and depressive symptoms were opposite in conflict events (with more I-talk indicating lower levels of symptoms).

Secondly, we tested whether markers of self-focus (greater proportion of I-talk and lower proportion of distancing) may begin to explain our earlier finding that greater episodic detail predicts depressive symptoms in turning point (but not conflict event) memory narratives. Results supported the role of self-focus: at both timepoints the positive relationship between greater episodic detail in the turning point and depressive symptoms was significant under conditions of high levels of I-talk, but not significant when I-talk is low. Moreover, the second measure of self-focus, linguistic distancing, confirmed this pattern: at both timepoints, the association between detail and depressive symptoms depended on low and medium levels of linguistic distancing, whereas when the proportion of distancing words were high the association is no longer significant. We did not find the same pattern for the conflict event, thus results provide preliminary support for the maladaptive role of greater detail uniquely in turning points when self-focus is exacerbated. It is important, however, to note that there were no significant longitudinal associations, and so conclusions are limited by the cross-sectional nature of the findings.

## General Discussion

The primary research aim was to first, determine whether we would replicate previous findings (Salmon et al., 2021) that greater total episodic (internal) detail (and not total external detail) in a turning point memory narrative predicted youth depressive symptoms concurrently and longitudinally; and second, to extend these findings by comparing the relationships for the turning point with those for a less self-relevant memory type (a conflict event narrative). Our secondary aim was to test our proposal that the heightened salience of the self while recalling a turning point plays a role, and to do so, we investigated linguistic markers of self-focused attention (greater proportion of I-talk and lower proportion of distancing language) within youth’s narratives (turning points and conflict events) in relation to detail and depressive symptoms.

Findings from Study A concurrently replicated our previous research, yet the longitudinal relationship between episodic detail and depressive symptoms turned out to be the reverse of the direction demonstrated in the earlier study (Salmon et al., 2021). Specifically, in the current study young people with higher depressive symptoms at Time 1 reported greater detail at follow-up. Aspects of our sample and methodology may have contributed to our failure to replicate greater detail predicting escalating depressive symptoms. For example, unexpectedly, depressive symptoms decreased across time, in contrast to research demonstrating that rates of depression tend to increase between 13 and 18 years, particularly for girls (Pfeifer & Allen, 2021). Moreover, the shorter six-months follow-up period of the current study may have captured young people’s symptoms at a less demanding time of their academic year compared to the previous study’s follow-up over one year. Nonetheless, in contrast to previous research (Biedermann et al., 2017; Söderlund et al., 2014), the current findings support the, at times, maladaptive role of remembering the past in greater detail (Kyung et al., 2016; Moscovitch et al., 2018; Salmon et al., 2021; Wang et al., 2018).

Consistent with our second prediction, the association between greater episodic detail and higher depressive symptoms was *unique* to turning point narratives and did not emerge for the conflict event memory narrative. Research conducted within a narrative identity theoretical framework (McLean et al., 2017, 2020) has shown that event type matters when it comes to associations with psychological wellbeing, and our findings support this idea. Further, recent research with young adults (Fozzard-Costigan et al., 2023) confirmed the unique concurrent relationship between episodic detail and depressive symptoms in turning points compared to other narratives of a critical life event (low point and high point). Turning points have been demonstrated to be more self-relevant than other narrative types (Grysman & Hudson, 2011), as they prompt for a life changing event and self-reflection on how that event has impacted the person and their life. In contrast, the current study did not ask participants to describe how the conflict event had changed them, thus prompting for experiences that were perhaps less connected to identity and sense of self (McAdams, 2001) and more episodic in nature. In support of this idea, we note that the young people’s narratives of conflict events, as compared to turning points, contained significantly more internal detail, less external detail, and less emphasis on self. We propose that heightened self-relevance and self-focus may begin to explain why the association between greater detail and higher depressive symptoms is found uniquely in turning point memory narratives.

We, and others (Kyung et al., 2016; Salmon et al., 2021; Wang et al., 2018), have suggested that excessive self-focus may provide an explanation for the association between greater episodic detail and depressive symptoms. Findings from Study A tentatively supported the role of self-focus, in that we found that this association was unique to the highly self-relevant turning point memory narrative. Subsequently, in Study B, we systematically tested whether linguistic markers of self-focused perspective (greater proportion of I-talk and lower proportion of distancing language) exacerbated the relationship between greater episodic detail and depressive symptoms. Results partially supported our proposal. Specifically, the concurrent association between greater detail and higher depressive symptoms at both time points was magnified under conditions of higher self-focused linguistic features (higher I-talk and lower distancing), although the longitudinal association was found to be non-significant. When turning points included high or medium amounts of I-talk (first-person pronouns) or were low in distancing (language focused on the self in the here and now), greater detail was associated with higher depressive symptoms. In contrast, when turning points contained low levels of I-talk or medium or high levels of distancing language, no significant association between episodic detail and depressive symptoms was found. These findings support, at least concurrently, the role of heightened self-focus in the turning point as a prerequisite for the association between greater episodic detail and depressive symptoms, as well as highlighting distancing as a buffering factor.

Why might elevated self-focused attention and episodic detail in the context of a turning point be problematic for some young people, whereas lessening the focus on the self (distancing) be helpful? Research suggests that recalling the past in detail when self-focused attention is high can produce negative affect, and taking a more distant perspective is, in comparison, a more effective emotion regulation strategy (Boucher & Scoboria, 2020; Orvell et al., 2021), particularly for people with depressive symptoms (Kross et al., 2012; Kross & Ayduk, 2009; Orvell et al., 2022a, b). Thus, one possibility is that negative affect may play a role in our research outcome that recalling detailed turning points with heightened self-focused attention predicts concurrent depressive symptoms. Nonetheless, the current findings are suggestive only and do not provide good evidence for causal mechanisms.

## Limitations and Future Directions

The present study offers insights into the importance of memory type and supports the, at times, maladaptive role of remembering highly self-relevant events in greater episodic detail. Limitations should be noted, in addition to those mentioned earlier. In particular, 81% of the sample identified as female, thus, results may not generalise to adolescent males and future examinations of possible gender effects are needed. Although the current and previous findings (Salmon et al., 2021) suggest that greater episodic detail and depressive symptoms may be reciprocally related, further longitudinal research is needed to address this possibility.

Although our findings offer preliminary support for the role of excessive self-focus in concurrent data, whether it plays a role in moderating the association between detail and escalating depressive symptoms across time needs clarification. Further, our understanding of the specific qualities of the turning point that render them unique could be delineated by removing the instruction to draw an implication for self (thereby increasing the similarity of the turning point and conflict event instructions). In addition, an important avenue of future research is to investigate whether associations between detail and depressive symptoms depend on the valence of the turning point (in particular whether the experience changed the person and their life for better or worse). Finally, causal relationships could be elucidated by experimental research in which, for example, self-focus is experimentally induced and the influence on narrative episodic detail and affect is assessed.

Effective interventions for young people at risk of escalating depression are relatively limited (Weisz et al., 2017), despite this age being a prime time to target cognitive biases (Lau & Pile, 2015). Our findings, that recalling detailed highly self-relevant memories may not be beneficial for young people with elevated depressive symptoms, contrasts with current autobiographical memory intervention approaches that seek to enhance recall of specific and detailed memories (Barry et al., 2021; Hallford et al., 2020) and moreover, suggests these approaches may at times be counterproductive. Thus, further research on greater episodic detail and the development and maintenance of adolescent depressive symptoms is of critical importance to inform best practice in interventions designed to address early signs of youth depression (Lee et al., 2019).

## Conclusions

The current study extends previous research to show, firstly in Study A, that uniquely for the turning point narrative, youth with depressive symptoms were more likely to recall greater episodic detail concurrently and six months later. In Study B, we showed that heightened self-focus (higher proportions of I-talk and lower linguistic distancing) in conjunction with greater episodic detail predicted greater depressive symptoms concurrently. The current research, therefore, raises the possibility that greater episodic detail in highly self-relevant memory narratives may be a marker of depressive symptoms in adolescents when self-focus is high or distancing is low.

## Data Availability

The data that support the findings of this study are available from the corresponding author, [LK], upon request.
